# Supplementary material for: Modeling glioblastoma heterogeneity as a dynamic network of cell states
Source: Mol Syst Biol. 2021 Sep 16;17(9):e10105. doi: 10.15252/msb.202010105 (PMC8444284; doi:10.15252/msb.202010105)
Supplement: Supplementary file 6 — Source Data for Figure 5 [file MSB-17-e10105-s004.zip › Figure5A_sourcedata/GSEA_3017/nosigdb_stateB.GseaPreranked.1622795021725/DALMO_BARCODENUMBER_UNPUB.html]

Details for gene set DALMO\_BARCODENUMBER\_UNPUB[GSEA]

|  || Dataset | state43017 |
| Phenotype | NoPhenotypeAvailable |
| Upregulated in class | na\_pos |
| GeneSet | DALMO\_BARCODENUMBER\_UNPUB |
| Enrichment Score (ES) | 0.7220383 |
| Normalized Enrichment Score (NES) | 4.1657486 |
| Nominal p-value | 0.0 |
| FDR q-value | 0.0 |
| FWER p-Value | 0.0 |
Table: GSEA Results Summary

  

Fig 1: Enrichment plot: DALMO\_BARCODENUMBER\_UNPUB      
 Profile of the Running ES Score & Positions of GeneSet Members on the Rank Ordered List

  

| PROBE | GENE SYMBOL | GENE\_TITLE | RANK IN GENE LIST | RANK METRIC SCORE | RUNNING ES | CORE ENRICHMENT || 1 | HIST1H4C |  |  | 0 | 1.373 | 0.0258 | Yes |
| 2 | HIST1H1B |  |  | 1 | 1.272 | 0.0497 | Yes |
| 3 | TOP2A |  |  | 3 | 1.079 | 0.0685 | Yes |
| 4 | RRM2 |  |  | 7 | 0.875 | 0.0804 | Yes |
| 5 | UBE2C |  |  | 11 | 0.802 | 0.0910 | Yes |
| 6 | TPX2 |  |  | 12 | 0.801 | 0.1060 | Yes |
| 7 | NUSAP1 |  |  | 13 | 0.801 | 0.1211 | Yes |
| 8 | HMGB2 |  |  | 14 | 0.786 | 0.1359 | Yes |
| 9 | NCAPG |  |  | 16 | 0.778 | 0.1490 | Yes |
| 10 | SPC25 |  |  | 17 | 0.763 | 0.1633 | Yes |
| 11 | CDCA2 |  |  | 19 | 0.751 | 0.1760 | Yes |
| 12 | ESCO2 |  |  | 20 | 0.742 | 0.1899 | Yes |
| 13 | TACC3 |  |  | 21 | 0.741 | 0.2038 | Yes |
| 14 | GTSE1 |  |  | 22 | 0.732 | 0.2176 | Yes |
| 15 | HJURP |  |  | 23 | 0.731 | 0.2313 | Yes |
| 16 | KIF23 |  |  | 24 | 0.721 | 0.2449 | Yes |
| 17 | ASPM |  |  | 26 | 0.695 | 0.2564 | Yes |
| 18 | KIF15 |  |  | 29 | 0.684 | 0.2663 | Yes |
| 19 | ARHGAP11A |  |  | 30 | 0.683 | 0.2791 | Yes |
| 20 | PCLAF |  |  | 31 | 0.681 | 0.2919 | Yes |
| 21 | SGO1 |  |  | 32 | 0.677 | 0.3046 | Yes |
| 22 | KPNA2 |  |  | 33 | 0.676 | 0.3173 | Yes |
| 23 | CENPF |  |  | 34 | 0.670 | 0.3299 | Yes |
| 24 | SGO2 |  |  | 36 | 0.664 | 0.3409 | Yes |
| 25 | KIF4A |  |  | 37 | 0.662 | 0.3533 | Yes |
| 26 | ANLN |  |  | 39 | 0.659 | 0.3642 | Yes |
| 27 | CENPE |  |  | 43 | 0.654 | 0.3720 | Yes |
| 28 | NUF2 |  |  | 44 | 0.651 | 0.3843 | Yes |
| 29 | PRR11 |  |  | 45 | 0.649 | 0.3965 | Yes |
| 30 | DLGAP5 |  |  | 46 | 0.647 | 0.4086 | Yes |
| 31 | KIFC1 |  |  | 47 | 0.645 | 0.4207 | Yes |
| 32 | DHFR |  |  | 49 | 0.638 | 0.4312 | Yes |
| 33 | NCAPG2 |  |  | 50 | 0.636 | 0.4432 | Yes |
| 34 | CENPA |  |  | 51 | 0.636 | 0.4551 | Yes |
| 35 | CDCA5 |  |  | 52 | 0.632 | 0.4670 | Yes |
| 36 | CCNA2 |  |  | 54 | 0.626 | 0.4773 | Yes |
| 37 | CKAP2L |  |  | 59 | 0.611 | 0.4827 | Yes |
| 38 | KIF2C |  |  | 66 | 0.591 | 0.4848 | Yes |
| 39 | SMC2 |  |  | 68 | 0.586 | 0.4943 | Yes |
| 40 | CDK1 |  |  | 69 | 0.585 | 0.5053 | Yes |
| 41 | H2AFX |  |  | 71 | 0.576 | 0.5147 | Yes |
| 42 | CKS1B |  |  | 72 | 0.575 | 0.5254 | Yes |
| 43 | CIP2A |  |  | 73 | 0.574 | 0.5362 | Yes |
| 44 | RACGAP1 |  |  | 75 | 0.572 | 0.5455 | Yes |
| 45 | FOXM1 |  |  | 76 | 0.570 | 0.5562 | Yes |
| 46 | MAD2L1 |  |  | 77 | 0.569 | 0.5669 | Yes |
| 47 | PLK1 |  |  | 78 | 0.567 | 0.5775 | Yes |
| 48 | MMP2 |  |  | 80 | 0.565 | 0.5866 | Yes |
| 49 | CKS2 |  |  | 83 | 0.558 | 0.5941 | Yes |
| 50 | AURKB |  |  | 89 | 0.548 | 0.5969 | Yes |
| 51 | PIF1 |  |  | 90 | 0.545 | 0.6072 | Yes |
| 52 | SPC24 |  |  | 91 | 0.544 | 0.6174 | Yes |
| 53 | CENPM |  |  | 92 | 0.544 | 0.6276 | Yes |
| 54 | CCNB1 |  |  | 93 | 0.544 | 0.6378 | Yes |
| 55 | NCAPH |  |  | 94 | 0.543 | 0.6480 | Yes |
| 56 | PBK |  |  | 95 | 0.540 | 0.6582 | Yes |
| 57 | SKA3 |  |  | 99 | 0.525 | 0.6636 | Yes |
| 58 | CDKN3 |  |  | 108 | 0.513 | 0.6612 | Yes |
| 59 | UBE2T |  |  | 110 | 0.512 | 0.6693 | Yes |
| 60 | CDCA3 |  |  | 111 | 0.511 | 0.6789 | Yes |
| 61 | DIAPH3 |  |  | 112 | 0.510 | 0.6885 | Yes |
| 62 | BUB1 |  |  | 113 | 0.508 | 0.6980 | Yes |
| 63 | TUBB4B |  |  | 114 | 0.507 | 0.7075 | Yes |
| 64 | BIRC5 |  |  | 116 | 0.504 | 0.7155 | Yes |
| 65 | KIF18B |  |  | 123 | 0.494 | 0.7158 | Yes |
| 66 | SPDL1 |  |  | 126 | 0.492 | 0.7220 | Yes |
| 67 | FANCD2 |  |  | 149 | 0.461 | 0.6977 | No |
| 68 | TK1 |  |  | 167 | 0.444 | 0.6805 | No |
| 69 | PIMREG |  |  | 169 | 0.443 | 0.6873 | No |
| 70 | MKI67 |  |  | 179 | 0.436 | 0.6820 | No |
| 71 | CDC20 |  |  | 184 | 0.431 | 0.6841 | No |
| 72 | MIS18BP1 |  |  | 186 | 0.430 | 0.6906 | No |
| 73 | H2AFZ |  |  | 216 | 0.405 | 0.6547 | No |
| 74 | POC1A |  |  | 234 | 0.390 | 0.6365 | No |
| 75 | PARPBP |  |  | 240 | 0.387 | 0.6363 | No |
| 76 | HMGN2 |  |  | 243 | 0.386 | 0.6405 | No |
| 77 | CCNB2 |  |  | 250 | 0.379 | 0.6386 | No |
| 78 | PLK4 |  |  | 255 | 0.374 | 0.6397 | No |
| 79 | CENPW |  |  | 267 | 0.366 | 0.6300 | No |
| 80 | ANP32E |  |  | 280 | 0.358 | 0.6187 | No |
| 81 | RAMP1 |  |  | 285 | 0.357 | 0.6194 | No |
| 82 | CENPN |  |  | 309 | 0.343 | 0.5913 | No |
| 83 | DEK |  |  | 316 | 0.341 | 0.5887 | No |
| 84 | PHF19 |  |  | 319 | 0.339 | 0.5921 | No |
| 85 | LRR1 |  |  | 410 | 0.304 | 0.4627 | No |
| 86 | PTTG1 |  |  | 413 | 0.302 | 0.4653 | No |
| 87 | ITGA7 |  |  | 454 | 0.291 | 0.4108 | No |
| 88 | MZT1 |  |  | 456 | 0.291 | 0.4147 | No |
| 89 | IDS |  |  | 465 | 0.289 | 0.4081 | No |
| 90 | APLP1 |  |  | 480 | 0.285 | 0.3925 | No |
| 91 | DKK3 |  |  | 549 | 0.273 | 0.2955 | No |
| 92 | GPC1 |  |  | 651 | 0.256 | 0.1487 | No |
| 93 | LONRF2 |  |  | 677 | 0.251 | 0.1158 | No |
| 94 | NEAT1 |  |  | 714 | -0.308 | 0.0676 | No |
Table: GSEA details [plain text format]

  

Fig 2: DALMO\_BARCODENUMBER\_UNPUB: Random ES distribution      
 Gene set null distribution of ES for **DALMO\_BARCODENUMBER\_UNPUB**
